# A Novel Approach for Purification and Selective Capture of Membrane Vesicles of the Periodontopathic Bacterium, *Porphyromonas gingivalis*: Membrane Vesicles Bind to Magnetic Beads Coated with Epoxy Groups in a Noncovalent, Species-Specific Manner

**DOI:** 10.1371/journal.pone.0095137

**Published:** 2014-05-15

**Authors:** Ryoma Nakao, Kenji Kikushima, Hideo Higuchi, Nozomu Obana, Nobuhiko Nomura, Dongying Bai, Makoto Ohnishi, Hidenobu Senpuku

**Affiliations:** 1 Department of Bacteriology I, National Institute of Infectious Diseases, Tokyo, Japan; 2 Department of Science, The University of Tokyo, Tokyo, Japan; 3 Faculty of Life and Environmental Sciences, University of Tsukuba, Ibaraki, Japan; 4 Department of Gerodontology, Graduate school of Tokyo Medical and Dental University, Tokyo, Japan; University of California, Merced, United States of America

## Abstract

Membrane vesicles (MVs) of *Porphyromonas gingivalis* are regarded as an offensive weapon of the bacterium, leading to tissue deterioration in periodontal disease. Therefore, isolation of highly purified MVs is indispensable to better understand the pathophysiological role of MVs in the progression of periodontitis. MVs are generally isolated by a conventional method based on ultracentrifugation of the bacterial culture supernatant. However, the resulting MVs are often contaminated with co-precipitating bacterial appendages sheared from the live bacteria. Here, we report an intriguing property of *P. gingivalis* MVs–their ability to bind superparamagnetic beads coated with epoxy groups (SB-Epoxy). Analysis of fractions collected during the purification revealed that all MVs of five tested *P. gingivalis* stains bound to SB-Epoxy. In contrast, free fimbriae in the crude MV preparation did not bind to the SB-Epoxy. The SB-Epoxy-bound MVs were easily dissociated from the SB-Epoxy using a mild denaturation buffer. These results suggest that the surface chemistry conferred by epoxy on the beads is responsible for the binding, which is mediated by noncovalent bonds. Both the structural integrity and purity of the isolated MVs were confirmed by electron microscopy. The isolated MVs also caused cell detachment from culture dishes at a physiologically relevant concentration. Assays of competitive binding between the SB-Epoxy and mixtures of MVs from five bacterial species demonstrated that only *P. gingivalis* MVs could be selectively eliminated from the mixtures. We suggest that this novel approach enables efficient purification and selective elimination of *P. gingivalis* MVs.

## Introduction

Among the various Gram-negative anaerobes that reside within the subgingival pockets, *Porphyromonas gingivalis* is regarded as a keystone pathogen in the development of periodontal diseases, due to its ability to orchestrate inflammation while being a minor constituent in the community of periodontal pockets [1]. Transient bacteremia occurs even after tooth brushing in periodontally healthy individuals [2] and the incidence and magnitude of bacteremia after scaling is significantly higher in periodontitis patients than in gingivitis patients and healthy control individuals [3]. For that reason, periodontitis patients are at risk for a wide range of bacteria-related systemic diseases based on persistent inflammation. Recent reports from epidemiological studies [4], [5] as well as in vitro and animal model experiments [6]–[8] have shown an association between periodontitis and systemic inflammatory diseases such as diabetes mellitus, cardiovascular disease, and atherosclerosis. Therefore, development of a novel remedy against periodontal diseases would have a significant impact on improving general public health.

Both Gram-negative and Gram-positive bacteria produce and release spherical, microstructural bodies called membrane vesicles (MVs) that range in size from 10 to 300 nm in diameter [9]. MVs contribute to a pathogen’s potential through toxin export [10]–[12] and adherence to eukaryotic cells [13]. As with other bacteria, *P. gingivalis* MVs have also been regarded as offensive weapons leading to tissue deterioration in periodontal disease [14]. MVs carry a wide range of virulence factors such as LPS, gingipains Rgps and Kgp, hemagglutin HagA, carboxypeptidase CPG70, peptidylarginine deiminase PAD, hemin-binding protein HBP35, and fimbrial proteins FimA and FimD; notably, periodontal tissue destruction mediated by gingipain-laden MVs has been suggested [15]. On the other hand, MVs (of *Neisseria meningitidis*) are also used for vaccination purposes to protect against bacterial pathogens in human [16]. Recently, the crucial role of *P. gingivalis* MVs in mucosal immunogenicity was shown by an in vivo study [17], suggesting the potential of MVs as non-replicating mucosal immunogens for periodontal disease vaccines.

Several methods have been used for the isolation of bacterial MVs from culture supernatants. Precipitation using ammonium sulfate [18] or ultracentrifugation [19] are the most common procedures. However, these methods do not yield sufficiently pure material for experimental use, because bacterial appendages such as fimbriae and flagella also co-precipitate with MVs of *Escherichia coli* [20] and *Pseudomonas aeruginosa* [21] during ultracentrifugation under the commonly used conditions. In general, *P. gingivalis* MVs are conventionally isolated from culture supernatant using a combination of membrane filtration and ultra-centrifugation [17], [22], [23]. In our previous study, fimbriae liberated from whole cells of *P. gingivalis* also appeared to co-precipitate with the MVs after ultracentrifugation [15]. Further purification using density-gradient centrifugation is an option, however, the procedure is relatively complicated and time-consuming. Therefore, development of novel methodology that enables purification of highly purified MVs or selective capture of the MVs is highly desired to enable a better understanding of the involvement of MVs in immunopathology and vaccinology of periodontal diseases.

In this article, we describe a simple and efficient approach to obtain highly purified MVs of *P. gingivalis* by exploiting the specific binding of MVs to superparamagnetic beads coated with epoxy groups (SB-Epoxy). Separation technology using SBs has been widely applied for purifying proteins, nucleic acids, and cells. The main advantages of this magnetic separation technique are due to their superparamagnetic properties, which result in easy handling, negligible sample loss, and superior reproducibility. We present here the technique’s utility for purifying MVs, and the structural and biological integrity of the resulting MVs. We also report that only *P. gingivalis* MVs are successfully eliminated from mixtures of MVs from different bacterial species. The applicability of this novel approach for both experimental and clinical settings is discussed.

## Materials and Methods

### 1. MV Preparation by the Conventional Method

*P. gingivalis* strain ATCC 33277 was the primary strain used in this study, as many fimbriae are found in crude MV preparation. Strains FDC 381, W50, YH522, and W83, were also used in some experiments. All strains of *P. gingivalis* were maintained under anaerobic conditions as described previously [24]. MVs were prepared from a culture of *P. gingivalis* collected at early stationary phase, described previously [17]. In brief, a two-day bacterial culture was centrifuged at 7,190×*g* for 30 min at 4°C. The supernatant was collected and filtered through a PVDF membrane with pore size 0.22 µm. The flow-through was collected and further ultra-centrifuged at 100,000×*g* for 3 h at 4°C. The resulting pellet was resuspended in 20 mM Tris-Cl (pH 8.0) and used as “crude MVs”. We also isolated crude MVs from eleven other strains of two oral bacteria and four non-oral bacteria as follows: two *Aggregatibacter actinomycetemcomitans* strains Y4 (our collection) and ATCC 29522 (our collection), two *Fusobacterium nucleatum* strains ATCC 23726 (our collection) and #20 (a gift from Kazuyuki Ishihara at Tokyo Dental College); three strains of *Escherichia coli* [one K-12 strain KP7600 (NIG collection, Japan) and two uropathogenic *E. coli* strains P15 and RC4 (our collection)]; one strain of *N. meningitidis* H44/76 [25]; two strains of *Pseudomonas aeruginosa* PAO1-Tokai (our collection) and PAO1-Holloway (our collection); and one *Clostridium perfringens* strain 13 (our collection). Crude MVs from *A. actinomycetemcomitans* were isolated by using the same isolation method used for crude *P. gingivalis* MVs. Crude MVs from species other than *P. gingivalis* and *A. actinomycetemcomitans* were prepared in the manner except for using PVDF membranes with pore size 0.45 µm. PVDF filters with pore size 0.22 µm were used for filtration of MVs of *P. gingivalis* and *A. actinomycetemcomitans*, as the diameter of these cells is smaller than that of the other species used in this study. We used the Bradford assay as a protein-based quantification method to determine the amount of purified MV.

### 2. Further Purification of MVs using SB Technology

We previously observed that MVs prepared by ultracentrifugation of culture supernatants were contaminated with bacterial appendages such as fimbriae, as the bacterial appendages co-precipitate with the MVs [15]. To eliminate the contaminating bacterial appendages, we exploited the binding affinity between MVs and superparamagnetic beads (SBs) to further purify the MVs. Dynabeads M-270 epoxy (SB-Epoxy) (Life Technologies, Carlsbad, CA) were mainly used for the purpose. We also used two other SBs of the same bead size (2.8 µm in diameter) but with different surface chemistries, Dynabeads M-270 amine (SB-NH_2_) (Life Technologies) and Dynabeads M-270 carboxylic acid (SB-COOH) (Life Technologies) to compare the binding properties of MVs of *P. gingivalis*. Five µg of crude MV preparation from *P. gingivalis* was resuspended in 100 µl of 20 mM Tris-Cl (pH 8.0) (final concentration of MVs: 500 ng/ml) and incubated with 20 mg (wet weight) of the SBs for 14 hours at 4°C on a rotator. After incubation, the SB-MV conjugates were collected and washed five times with PBS to remove unbound components. To dissociate the bound components from the SBs, the beads were incubated with mild denaturation buffer containing urea at a concentration of 2 M for 5 min at 15–24°C. Incubation with the urea buffer was repeated once. After the SBs were washed once again with PBS, the components still bound to the SBs even after urea treatment were further treated with PBS containing 4% CHAPS. The volume of all buffers used during the purification was fixed at 100 µl. For the experiments described under section “*2.4. SDS-PAGE and Western blot*”, 60 µl of each fraction were mixed with same volume of 2X SDS-PAGE loading buffer containing 2% SDS and 6% 2-mercaptoethanol. The rest (40 µl) of each fraction were individually dialyzed in a single batch against the same container of 20 mM Tris-Cl (pH 8) buffer to remove urea or CHAPS. The dialyzed MV samples were concentrated by ultrafiltration and reconstituted with 20 mM Tris-Cl (pH 8) buffer to a final volume of 40 µl. The reconstituted fractions and the crude MVs samples (500 ng/ml) were diluted 1∶10 with PBS and used for the experiments described in section “*2.5. Assays for cell detachment from cell culture wells*”.

In some experiments, SDS-PAGE loading buffer containing 2% SDS and 6% 2-mercaptoethanol was directly added to SBs to elute the components bound to the SBs. After incubation of the crude MVs with SBs, the beads were also treated with 1 to 4 M NaCl buffer or 50 mM Glycine buffer (pH 2.8), in order to examine whether any of these buffers could dissociate the components bound to the SBs. Only SB-Epoxy was regenerated by washing with PBS containing 2% SDS, followed by five washes with PBS. Further, the binding efficacy of MVs to regenerated SB-Epoxy was unaffected even after reuse at least 10 times (data not shown).

### 3. Electron Microscopy (EM) Analysis

The MV-SB-Epoxy conjugates were prepared by the same method described in section “*2. Further purification of MVs using SB technology*”. The resultant MV-SB conjugates were analyzed by scanning EM, S-5200 (HITACHI, Tokyo, Japan). Sample preparation for scanning EM was described previously [17]. The crude MVs and the samples fractionated from the crude MVs were analyzed by transmission EM, H-7650 (HITACHI). Samples for transmission EM were allowed to adhere to carbon-coated grids for 1 min at 15–24°C, then negatively stained with 2% uranyl acetate.

### 4. SDS-PAGE and Western Blot (WB)

Samples fractionated from MV preparations were analyzed by a standard protocol of SDS-PAGE using 12.5% polyacrylamide gels. After SDS-PAGE, some gels were visualized with staining using silver or Bio-Safe Coomassie (CBB, Bio-rad), and others were electroblotted onto PVDF membranes for WB analysis. Rabbit polyclonal antibody against a major fimbrial protein of *P. gingivalis* (anti-FimA antibody, a gift from Nobushiro Hamada at Kanagawa Dental University), a catalytic domain of arginine-gingipain A (anti-Rgp antibody) [26], and a hemin-binding protein 35 (anti-HBP35 antibody) [27], were used at 1∶1000, 1∶10,000, and 1∶5,000 dilutions, respectively. Mouse monoclonal antibody against an anionic lipopolysaccharide of *P. gingivalis* (anti-A-LPS antibody) [26] was used at 1∶1000 dilution. Horseradish peroxidase-labeled anti-rabbit IgG (GE Healthcare, Buckinghamshire, UK) and anti-mouse IgG (Life technologies) were used as the secondary antibody at 1∶200,000 and 1∶5,000 dilutions, respectively. Chemiluminescence was developed by ECLplus (GE Healthcare) and visualized by exposure on X-ray film.

### 5. Assays for Cell Detachment from Cell Culture Wells

An oral squamous epithelial cell carcinoma cell line, Ca9-22 [28], was maintained in RPMI1640 media supplemented with 10% heat-inactivated fetal bovine serum, penicillin (100 U/ml), and streptomycin (50 µg/ml) at 37°C in a 5% CO_2_ incubator. The effect of samples prepared from *P. gingivalis* on cell detachment was examined on confluent monolayers of Ca9-22 cells in 24-well culture plates. The cell monolayers were washed three times with PBS to wash out the residual fetal bovine serum in the media. Ten-fold diluted samples were prepared as described in section “*2. Further purification of MVs using SB technology*”. Two hundred µl of the diluted samples were added to the wells. The cells were incubated at 37°C in a 5% CO_2_ incubator for 1 hour. The wells were gently washed twice with 1 ml of PBS to remove detached cells. Changes in cell appearance were recorded after the two washes with PBS by an inverted-optical microscopy (CKX41, Olympus, Tokyo, Japan). Eventually, undetached cells were fixed with 200 µl of 70% methanol for 10 min, stained with 200 µl of 0.1% crystal violet for 20 min, and rinsed twice with 1 ml of distilled water. Crystal violet dye associated with cells was eluted with 500 µl of 100% ethanol. Amounts of detached Ca9-22 cells were quantified by absorbance at 595 nm.

### 6. Time-lapse Observation of Ca9-22 Cells

Ca9-22 cells were grown on a glass-bottom dish (CELLview cell culture dish, Greiner) coated with poly-L-lysine (Sigma-aldrich). The assay was performed soon after cells reached confluence. Following replacing the culture medium to Eagle’s balanced salt solution (EBSS) with or without purified MVs (20 µg/ml), phase contrast images of the cells were captured. The optical system consisted of an inverted-epifluorescence microscopy (IX71, Olympus) equipped with a phase objective (UPlanFl 40x, Olympus), a stage-top incubator (Tokai Hit) and an EMCCD camera (iXon DU-897E, Andor). The z-position of the objective was controlled by a piezo-electric actuator with an electrostatic capacity sensor (PI, Germany), which enabled us to obtain three-dimensional images and to overcome the drift in focus position during the long observation time.

### 7. Assay of Competitive Binding with SB-Epoxy using a Mixture of MV Preparations from Several Strains

We assayed the competitive binding of a mixture of crude MVs from several species with SB-Epoxy. Five µg each of crude MVs from *P. gingivalis* ATCC 33277, *E. coli* KP7600, *N. meningitidis* H44/76, *P. aeruginosa* PAO1-Tokai, and *C. perfringens* strain 13 were suspended together in 100 µl of 20 mM Tris-Cl (pH 8.0) containing 20 mg (wet weight) of the SB-Epoxy and incubated at 4°C for 14 hours on a rotator. After five washes with PBS, SDS-PAGE loading buffer was directly added to elute components bound to the SB-Epoxy. The wash and elution fractions collected during this procedure were analyzed by SDS-PAGE.

### 8. MALDI-TOF Mass Spectrometry and Peptide Mass Fingerprinting (PMF)

After SDS-PAGE, Coomassie brilliant blue stained protein bands were excised from the gels, and in-gel digestion with trypsin (Promega, Fitchburg, WI) was performed. The tryptic peptide samples were analyzed by an AB SCIEX TOF/TOF 5800 System (AB SCIFX, Foster City, CA). The MS data were analyzed by the Mascot Peptide Mass Finger Print search engine (Matrix Science, Inc., Boston, MA). Peptide mass tolerance for Mascot analysis was set at ±100 ppm, and one missed cleavage by trypsin and variable oxidation of methionine were allowed.

### 9. Statistical Analysis

Statistical analysis was performed using one-way analysis of variance (ANOVA) followed by the Dunnett's multiple comparison test. *P*-values of 0.05 or less were considered to indicate statistical significance.

## Results

### 1. P. gingivalis MVs Preferentially Bind to SB-Epoxy

**Figure 1:**
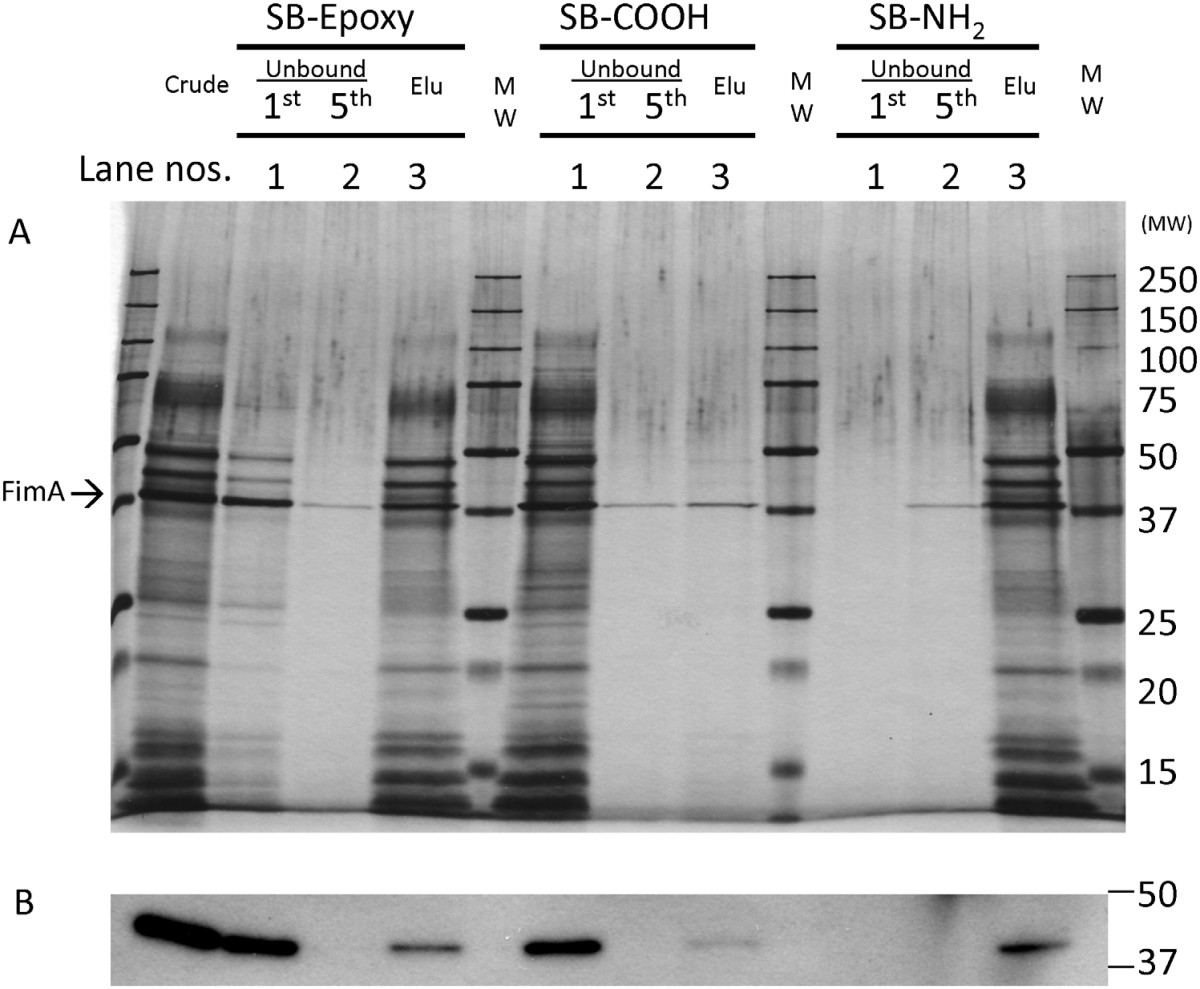
Binding of MVs of *P. gingivalis* to SBs. Binding of *P. gingivalis* MVs to SBs was examined by SDS-PAGE (A: silver staining, B: WB of FimA). Five µg of the starting material from conventional purification (Crude) was incubated with same amount (20 mg, wet weight) of three SBs with different surface chemistries (SB-Epoxy, SB-COOH, and SB-NH_2_). After incubating the crude MVs with SBs, unbound components were washed five times with PBS. The first and fifth unbound fractions are applied to the wells (Unbound, 1^st^ and Unbound, 5^th^, or lanes 1 and 2). Then bound components were eluted by addition of SDS-PAGE loading buffer (Elu or lane 3). “MW” denotes the molecular weight marker. The same volume (10 µl) of samples was applied to each well of the PAGE gel. (B) Identical samples to those used in (A) were used for the WB of FimA. The location of FimA is shown by an arrow.

During a course of experiments using SB-Epoxy and *P. gingivalis* MVs, we unexpectedly found that MVs of *P. gingivalis* appeared to bind to the beads after a 14-hour incubation at 4°C. In order to assess the binding properties of MVs to SBs, we chose three SBs with the following different surface chemistries–SB-NH_2_, SB-COOH, and SB-Epoxy–and examined the protein profiles of the fractions collected during purification (Fig. 1A). Physicochemical properties of the three SBs are shown in Table 1. We previously observed that *P. gingivalis* ATCC 33277 MVs prepared by ultracentrifugation contained many fimbriae [15]. Therefore, we chose strain 33277 for purification of *P. gingivalis* MVs and the crude MVs were used for the binding assay with the SBs. SDS-PAGE analysis revealed a typical protein profile of the 33277 MVs [15] in the lane of the “Crude” fraction (Fig. 1A). A major band on a silver-stained gel at 40 kDa was identified as the major fimbrial protein FimA by mass spectrometry (shown by the arrow in Fig. 1A) and WB (Fig. 1B). When SB-NH_2_ was used for this assay, both the MVs and the fimbriae bound to the SB-NH_2_, as few proteins were observed in the lanes of the unbound fractions (Unbound, 1^st^ and 5^th^). In contrast, with SB-COOH, both MVs and fimbriae bound minimally to the SB-COOH, as most of the starting materials (Crude) were washed out in the first unbound fraction (Unbound, 1^st^). Unlike SB-NH_2_ and SB-COOH, SB-Epoxy could bind MVs while most fimbriae were removed by washing, although a faint FimA signal was detected in the elution fraction even after extensive washing.

**Table 1:** Comparison of three different SBs with different surface chemistries.

	SB-Epoxy	SB-NH_2_	SB-COOH
**Sizes (diameter)**	2.8 µm	2.8 µm	2.8 µm
**Chemical formula of** **surfaces**	Epoxy group	Amino group	Carboxylic acidgroup
**Hydrophobicity** *	More hydrophobic thanSB-COOH	More hydrophobic thanSB-COOH	Extremelyhydrophilic
**Charge** *	Slightly positive	Positive	Negative

*The hydrophobicity and surface charge of each SB were determined by a contact angle and zeta potential analysis, respectively. Information about SBs is available upon request from the authors (Dynal Biotech Technical Support, unpublished data).

**Figure 2:**
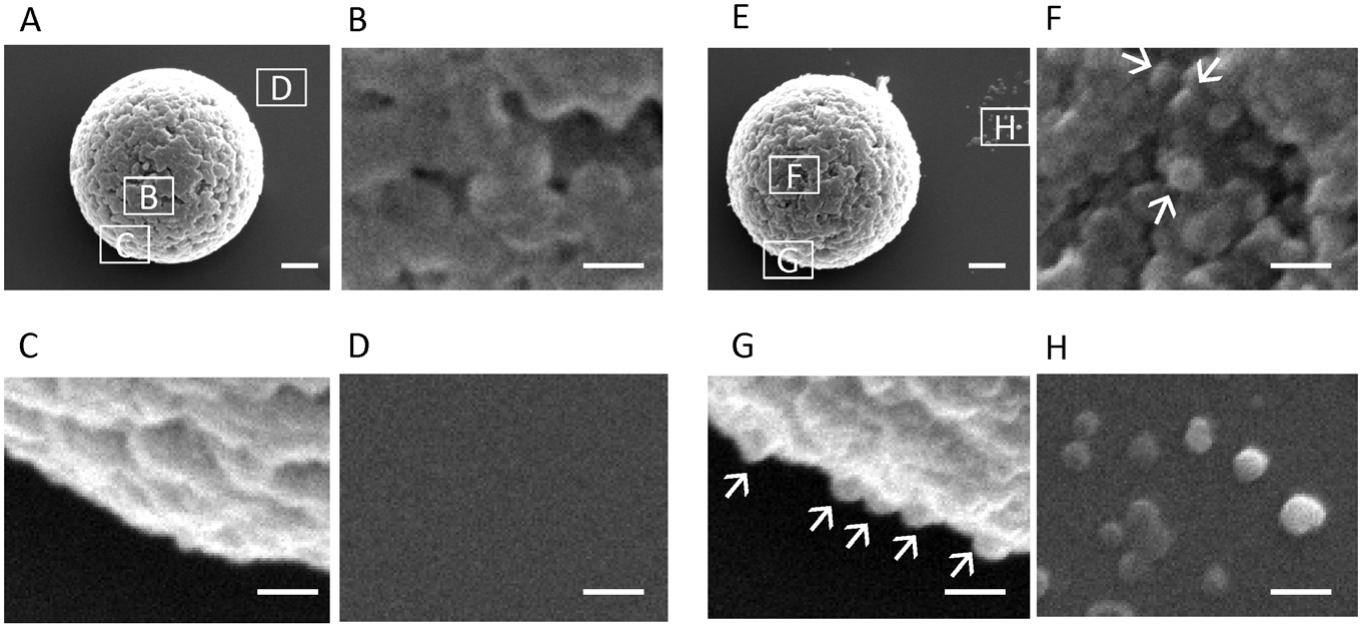
Electron microscopy of SB-Epoxy-MV conjugates. Electron micrographs of SB-Epoxy incubated with control buffer (A–D) or with the crude MVs (E–H) are shown. Areas of micrographs shown at higher magnification (B–D) and (F–H) are indicated in panels (A) and (E), respectively, by the corresponding boxed letters. MVs are shown by arrows. Bars in (A) and (E): 500 nm. Bars in (B–D) and (F–H): 100 nm.

The SB-Epoxy-MV conjugates were analyzed by scanning EM to directly observe binding (Fig. 2A–H). We clearly found MVs on the surface of the SB-Epoxy incubated with MVs but not on the SB-Epoxy incubated with control buffer. The dissociated MVs were also observed on the background behind the SB-Epoxy.

These data suggest that the binding of MVs with SBs is dependent on different surface chemistries conferred by different functional groups. Of note, the findings of the preferential and specific binding of MVs to SB-Epoxy suggested that these beads could be employed to improve the purification of *P. gingivalis* MVs. Therefore, in the present study we have focused on investigating the binding between the MVs and SB-Epoxy.

### 2. Isolation of MVs with High Purity and Structural Integrity using SB Technology

**Figure 3:**
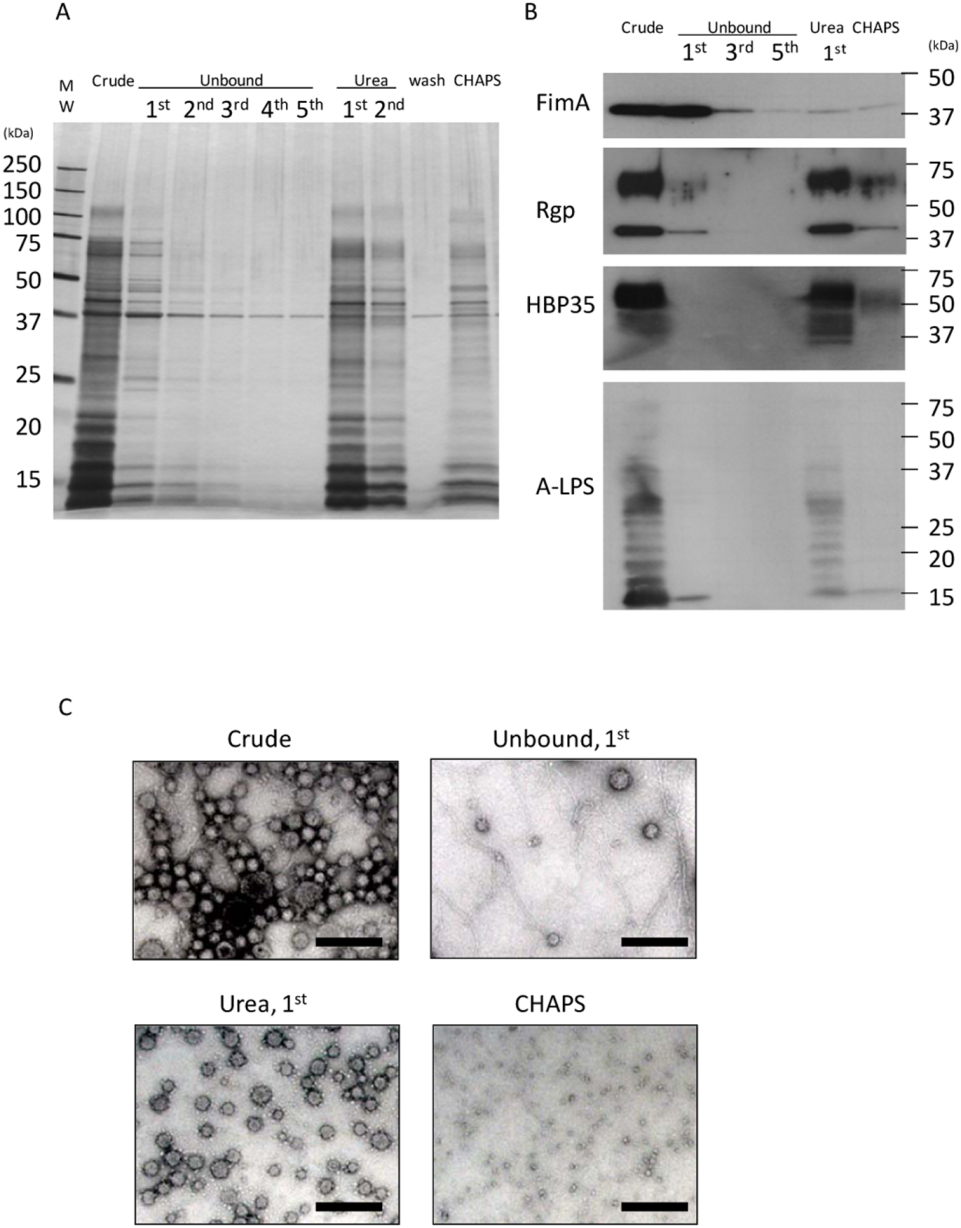
Isolation of highly purified MVs using SB-Epoxy. MV purification was performed by incubating SB-Epoxy with the crude MVs. Five µg of the starting material from conventional purification (Crude) was incubated with 20 mg (wet weight) of SB-Epoxy. After incubating the crude MVs with SBs, unbound components were washed five times with PBS (Unbound). Subsequently, bound components were eluted twice with a mild denaturation buffer containing 2 M urea (Urea). After one wash with PBS (Wash), the components still bound to the SB-Epoxy even after urea treatment were treated with 4% CHAPS buffer (CHAPS). “MW” denotes the molecular weight marker. (A) Each fraction was analyzed by silver staining. The same volume (10 µl) of samples was applied to each well of a PAGE gel. (B) The same sample set was used for WBs using anti-FimA, Rgp, HBP35, and A-LPS. In the WB of Rgp, diffuse bands around 60–70 kDa and relatively sharp bands around 40 kDa are observed, probably due to the presence of both glycosylated and non-glycosylated forms of Rgps in the MVs, respectively. HBP35 also appears as diffuse bands because of modification of the HBP35 protein with different-sized glycans. A typical ladder pattern is observed in WB detecting A-LPS. (C) The contents of each fraction was examined by transmission EM. Bars: 200 nm.

We tried to establish the conditions under which MVs would dissociate from SB-Epoxy (Fig. 3A and B). Similar to the results shown in Fig. 1A and B, most of the fimbriae were eliminated after five washes, with only a small amount of fimbriae remaining. First, the SB-Epoxy-MV conjugates were treated with either low pH buffer or high salt buffer. However, the MVs did not dissociate at all from SB-Epoxy (data not shown). Second, we treated the SB-Epoxy-MV conjugates with buffer containing different concentrations of urea ranging from 1 M to 8 M and found that greater than 2 M urea enables efficient dissociation of binding (data not shown). The fraction eluted by 2 M urea buffer contained major MV proteins such as gingipains and HBP35 as well as A-LPS (Fig. 3A and B) whose structure is characteristic of *P. gingivalis* [29]. These SDS-PAGE data showed good agreement with the transmission EM data where the fraction eluted with urea buffer (Urea, 1^st^) contained very little fimbriae, while fimbriae were abundantly detected in the starting materials (Crude) and in the first unbound fraction (Unbound, 1^st^), but not in the subsequent fractions (Fig. 3C). Consequently, we succeeded in isolating highly purified MVs that maintained their structural integrity. In contrast, there were no morphologically intact MVs in the fraction eluted by a detergent, 4% CHAPS (CHAPS), because the MV lipid bilayer was destroyed by CHAPS.

### 3. Detachment of Epithelial Cells from Cell Culture Wells Induced by the Highly Purified MVs

**Figure 4:**
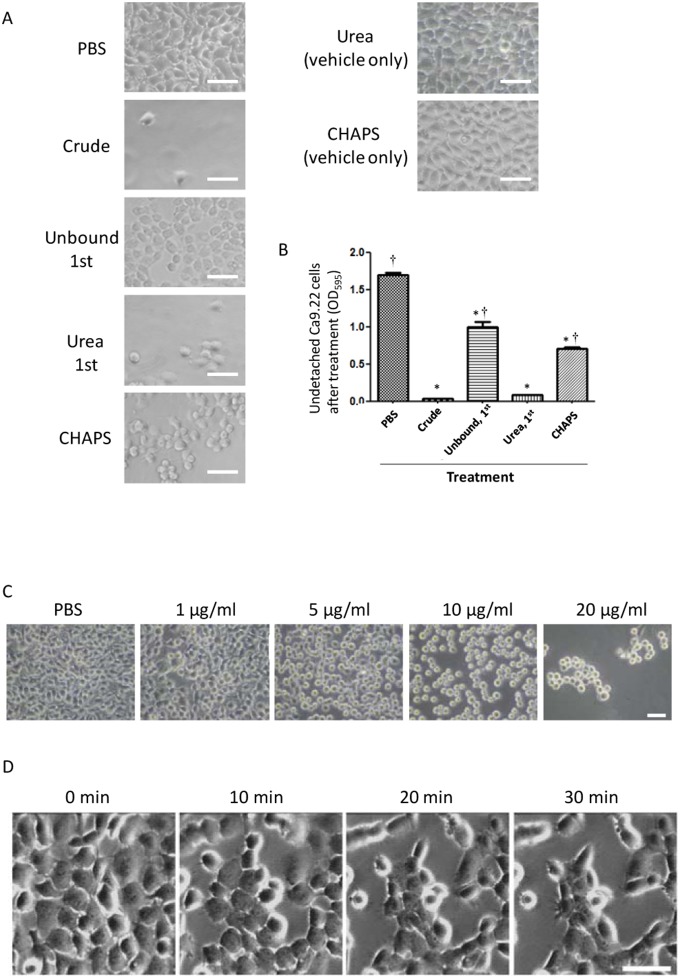
Effect on cell detachment of the crude MVs and SB purification fractions. Cell detachment activity of samples fractionated using SB-Epoxy was investigated using Ca9.22 cell monolayers. (A) The cell monolayers were treated with PBS only (PBS), crude MV preparation (Crude), first unbound fraction (Unbound, 1^st^), first urea fraction (Urea, 1^st^), and CHAPS fraction (CHAPS). In order to confirm complete removal of urea and CHAPS, the monolayers were also treated with dialyzed urea buffer [Urea (vehicle only)], and dialyzed CHAPS buffer [CHAPS (vehicle only)]. Bars: 50 nm. (B) The amount of detached cells was determined by crystal violet staining and measuring absorbance at 595 nm (mean ± SD), assayed in triplicate. *P<0.05, compared to PBS-treated cells. ^†^P<0.05, compared to cells treated with the crude MV preparation (Crude). (C) Cell appearances after treatment with PBS control (PBS) and PBS containing MVs at concentrations of 1, 5, 10, and 20 µg/ml are shown. After treatment with different concentrations of MVs, micrographs of the treated cells were taken. Bar: 50 µm. (D) Time-lapse images of Ca9.22 cells in the presence of the SB-purified MVs. Morphological changes of Ca9.22 cells are shown. Loss of focal adhesion and cell-cell adhesion resulted in cell rounding, followed by detachment from the culture wells. The images were acquired at intervals of 30 sec for 30 min immediately after addition of MVs [See Video S1 with S2 (movie of cells in the absence of MVs)]. Bar: 50 µm.

In a previous study, we found that *P. gingivalis* MVs caused detachment of epithelial cells in a gingipain-dependent manner [15]. To assess whether the MVs purified using SB-Epoxy are biologically intact, we performed a cell detachment assay using the crude MVs and the fractions from SB-Epoxy purification (Fig. 4). Following treatment with each sample, cell detachment activity was evaluated by staining undetached cells as well as microscopic observation (Fig. 4A and 4B). The strongest activity was in the starting material (Crude) and comparable activity was seen in the first urea-eluted fraction (Urea, 1^st^). Significantly lower activities were detected in the first unbound fraction (Unbound, 1^st^) and the CHAPS-eluted fraction (CHAPS) as compared with the starting material (Crude). To assess whether the dialysis was effective for complete removal of urea or CHAPS, the vehicle alone (2 M urea or 4% CHAPS without the MV sample) was dialyzed and used for the detachment assay [Urea (vehicle only) and CHAPS (vehicle only)]. Our findings confirmed that the dialyzed vehicle did not affect cell morphology or detachment (Fig. 4A). These results showed good agreement with the EM findings (Fig. 3C) where we observed a small amount of MVs in the “Unbound, 1^st^” fraction and the remains of destroyed MVs in the “CHAPS” fraction. We also observed a dose-dependent effect of the purified MVs on cell detachment (Fig. 4C). Time-lapse observation after addition of the purified MVs demonstrated that disruption of cell-cell adhesion and focal adhesion occurred swiftly (Fig. 4D, and Video S1 and S2).

### 4. Alternative Approach using SBs for Selective Capture of MVs of P. gingivalis

**Figure 5:**
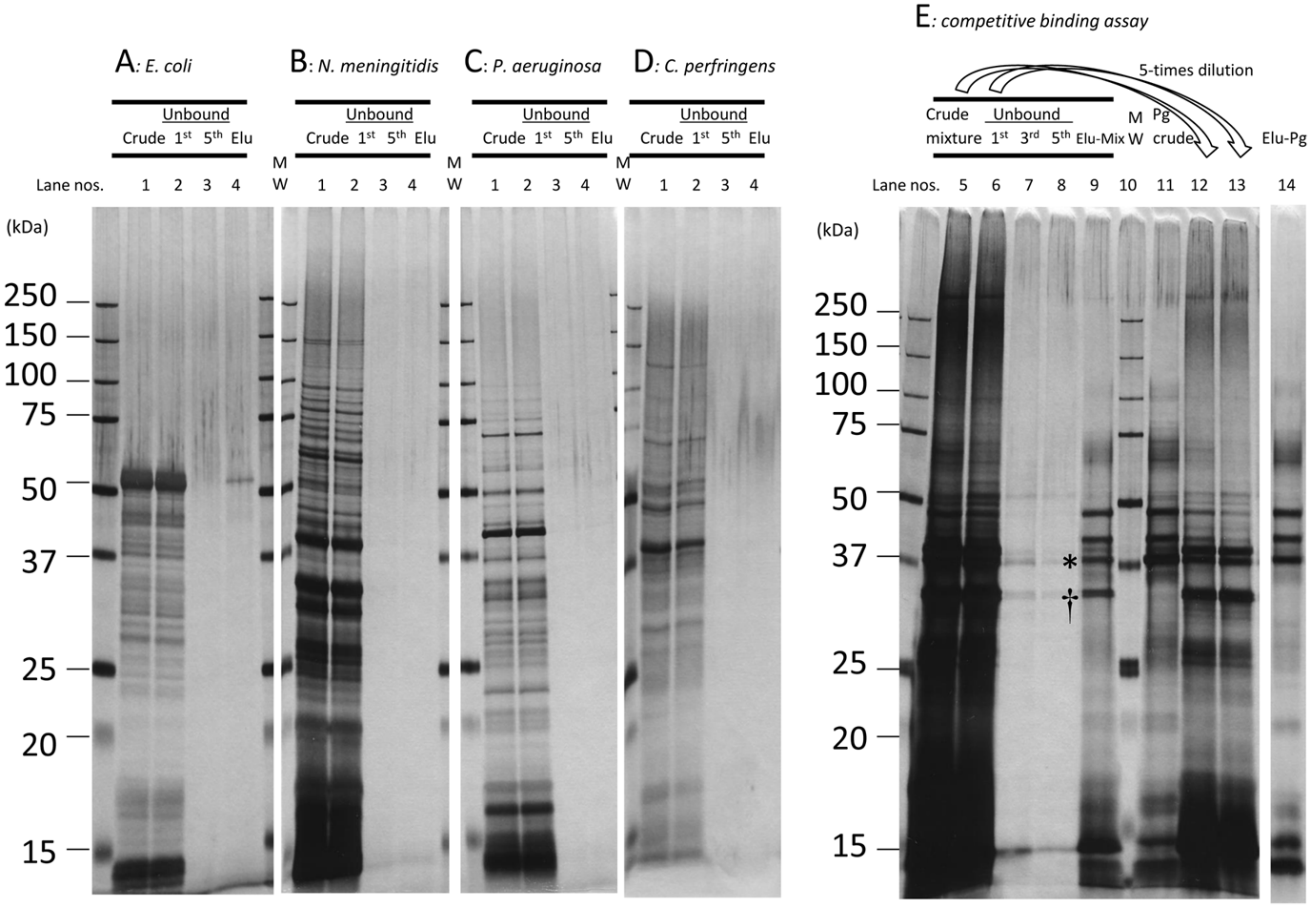
Binding to SB-Epoxy of MVs from different bacterial species. Shown are results of the assays using crude MV preparations from the following five different species: *E. coli* KP7600, *N. meningitidis* H44/76, *P. aeruginosa* PAO1 Holloway, *C. perfringens* strain 13, and *P. gingivalis* ATCC 33277. (A-D) Crude MVs from a single strain was incubated with the SB-Epoxy. Unbound components were collected in five washes and bound components were eventually eluted by SDS-PAGE loading buffer. Lanes denoted “1” are the starting material from conventional purification (Crude). Lanes denoted “2” are the first unbound fractions (Unbound, 1^st^). Lanes denoted “3” are the fifth unbound fractions (Unbound, 5^th^). Lanes denoted “4” are the elution fractions (Elu). (A: *E. coli* KP7600, B: *N. meningitidis* H44/76, C: *P. aeruginosa* PAO1-Tokai, D: *C. perfringens* strain 13). (E) Crude MVs from the five species were mixed with SB-Epoxy. The selective elimination of *P. gingivalis* MVs from the mixture is shown. Lanes 5 &12: the input of mixtures of crude MVs (Crude mixture). Lanes 6 & 13: the first unbound fractions (Unbound, 1^st^). Lanes 12 & 13: five-fold diluted samples of lanes 5 & 6, respectively. Lanes 7, 8, & 9: the third unbound fraction (Unbound, 3^rd^), the fifth unbound fraction (Unbound, 5^th^), and the elution fraction (Elu-Mix) from the mixture of four different MV preparations, respectively. Lane 10: the molecular weight marker. Lane 11: the crude MVs of *P. gingivalis* (Pg, Crude). Lane 14: the elution fraction from crude MVs of *P. gingivalis* only (Elu-Pg). The same volume (10 µl) was applied in each well of each PAGE gel. *P. gingivalis* FimA is denoted by an asterisk (*). *N. meningitidis* PorB is denoted by a dagger (†).

To examine whether *P. gingivalis* MVs bind to SB-Epoxy in a species-specific manner, we assessed whether MVs of other *P. gingivalis* strains also could bind to the SB-Epoxy and whether MVs derived from eleven strains of the other six species (including two other periodontopathic species) could bind to the SB-Epoxy. We confirmed that MVs isolated from other 4 strains of *P. gingivalis* as well as strain 33277 could bind to SB-Epoxy, although the binding capacities of these MVs depend on the individual strains (Fig. S1). On the contrary, SB-Epoxy bound extremely poorly to MVs of all the bacterial species tested except *P. gingivalis*. Results of one or two representative strain(s) of each species are shown (Fig. 5A–D and Fig. S2). To examine the applicability of this approach for selective capture of *P. gingivalis* MVs, we performed a competitive binding assay using SB-Epoxy and mixtures of crude MVs from several strains (Fig. 5E). Most of the crude MV preparation proteins (lanes 5 and 12, Crude mixture) were washed out in the first unbound fraction (lanes 6 and 13, Unbound 1^st^). A comparison of the protein profile of the elution fraction from the MV preparation mixtures (lane 9, Elu-Mix) with that of the *P. gingivalis* MV preparation preparation input (lane 11, Pg Crude) showed that the protein profile of the elution fraction (lane 9) was similar to that of the *P. gingivalis* crude MVs (lane 11), except for a decreased FimA band (denoted by asterisk) and unusual 34-kDa band (denoted by dagger). Furthermore, the protein profile in the lane 9 was nearly identical to that of the elution fraction from the *P. gingivalis* crude MVs (lane 14), except for only the 34-kDa band. The presence of *P. gingivalis* MVs in the elution fraction was also confirmed by WB using antiserum against *P. gingivalis*-specific A-LPS (data not shown). These data showed that *P. gingivalis* MVs could be selectively captured by specific binding to SB-Epoxy. The band at 34 kDa observed in the elution fraction was identified as PorB derived from *N. meningitides* by Mass spectrometry analysis. In order to focus on the association between *P. gingivalis* MV and neisserial PorA, we designed another competitive binding assay using MV preparations of two species: *P. gingivalis* and *N. meningitidis* (Fig. S3). In this assay, we again observed the decreased FimA band (denoted by an asterisk) and the 34-kDa band (PorB) (denoted by a dagger) in the elution fraction. These data demonstrated that SB-Epoxy selectively bound to *P. gingivalis* MVs and PorB was associated with the *P. gingivalis* MVs.

## Discussion

In the present study, SDS-PAGE and EM analysis revealed the preferential binding of *P. gingivalis* MVs to SB-Epoxy. The SB-Epoxy-bound MVs were efficiently dissociated from the SBs by treatment with a buffer containing 2 M urea. However, neither low pH buffer nor high salt buffer affected binding. In EM analysis, we clearly observed MVs on the surface of the SB-Epoxy. We also observed dissociated MVs on the background behind the SB-Epoxy, probably because the electrostatic force driven by the cationic poly-L-lysine, pre-coated on the glass surface, attracted the MVs to the beads. These data suggest that the major force holding these macromolecules together is dependent on noncovalent bonds, but not merely electrostatic attractions. In general, hydrophobic groups in a protein tend to cluster in the interior of the molecule and are buried on the inside of the protein in the absence of urea. It is widely accepted that conformational change of a protein occurs in the presence of urea and that urea preferentially interacts with the hydrophobic groups. For SB-Epoxy, the epoxy coating treatment on the SBs might confer surface hydrophobicity on the SBs, due to the intrinsic hydrophobicity of the epoxy group (Table1). We therefore speculate that the binding between MVs and SB-Epoxy might be via hydrophobic interactions; urea’s action on hydrophobic interactions triggers a conformational change in proteins on the surface of MVs, eventually leading to dissociation of MVs from SB-Epoxy. However, a definitive conclusion regarding the interaction mechanisms between SBs and MVs or fimbriae requires further investigation. On the other hand, *P. gingivalis* fimbriae preferentially bound to SB-NH_2_ but not to SB-COOH or SB-Epoxy. Both MVs and fimbriae bound to SB-NH_2_ were eluted by addition of low pH buffer or high salt buffer (data not shown), suggesting that the binding of both fimbriae and MVs to SB-NH_2_ might be through electrostatic attraction.

For the competitive binding assays, we chose four Gram-negative bacterial species with different LPS structures. The structure of *E. coli* LPS, which has been most extensively studied, is composed of a hydrophobic domain known as lipid A, a core oligosaccharide, and a long O- polysaccharide [30]. *P. gingivalis* possesses two different types of LPS. One type, the anionic polysaccharide-LPS termed A-LPS, is a unique LPS containing a phosphorylated branched mannan [31]. *N. meningitidis* LPS has a shorter carbohydrate chain called lipooligosaccharide, which is antigenically and structurally similar to carbohydrates present in human glycosphingolipids [32]. *P. aeruginosa* has two different LPS moieties called A-band (a short chain and neutral in charge) and B-band (a longer chain and negatively charged) [33]. We also used the MVs from *C. perfringens*, as a representative species of Gram-positive bacteria that do not have an outer membrane. Consequently, MVs prepared from all species tested except *P. gingivalis* did not bind to SB-Epoxy at all (Fig. 5A–D and Fig. S2), suggesting that the preferential binding depends on a cell-surface property conserved among *P. gingivalis* strains. It is therefore interesting to compare the surface chemistries of MVs isolated from different species. In conclusion, the novel approach described here using SB-Epoxy provides more selective and time-saving purification of *P. gingivalis* MVs compared to other conventional methods, such as density gradient centrifugation. These findings also suggest that the SB-Epoxy-based technique may be clinically applicable for eliminating *P. gingivalis* MVs that are released into the extracellular milieu, such as in periodontal pockets or the circulatory system.

We confirmed that cell detachment was strongly induced by highly purified MVs (Fig. 4 and Video 1 and 2). On the other hand, cell detachment activity was still somewhat high in the fraction eluted using CHAPS, suggesting that the MVs might have enhanced the stability of gingipains and protected their activity from CHAPS treatment, in consistent with previous reports [34], [35]. Purified MVs at concentrations of greater than 5 µg/ml clearly caused cell detachment. In our previous studies, we estimated that recovery of MVs from two-day culture supernatant of ATCC 33277 was approximately 10 µg per 1 ml of culture supernatant, according to our calculation by the Bradford method [15]. Taken together, these data suggest that a physiologically relevant concentration of MVs may cause destruction of periodontal tissues.

Results from both SDS-PAGE (Fig. 1) and EM (Fig. 2) demonstrated that most of the fimbriae in the crude MVs were not associated with SB-Epoxy, as they were eventually detected in the unbound fractions. However, we still detected a FimA signal in the elution fraction even after extensive washing of the beads. Similar to this result, a FimA signal was also detected in the MVs purified by using a combination of ultracentrifugation and density gradient separation (data not shown). We therefore suggest that fimbriae tightly bind to MVs or that fimbriae is one of the major components of MVs.

In the binding assay using SB-NH_2_, the WB of FimA showed that free fimbriae in the starting materials were not collected in unbound fractions, unlike the case of SB-Epoxy. Furthermore, the amount of FimA protein in the elution fraction was much lower than that in the crude MV starting material. These findings suggest that fimbriae did not dissociate from SB-NH_2_ even after addition of elution buffer containing 2% SDS.

Several vaccine projects targeting *P. gingivalis* infection are on-going in different research groups. In the future, we expect *P. gingivalis* infection to be controlled by vaccine protection. *P. gingivalis* MVs are a feasible immunogen for use as a periodontal disease vaccine, because a range of virulence factors are enriched in the MVs [15], which have been shown to elicit strong mucosal immune responses in a mouse model [17]. However, MVs used in these mouse experiments were isolated by the conventional purification method, that is, free fimbriae in the MV preparation were not removed. In the present article, the novel purification method using SB-Epoxy could improve the purity of MVs thanks to removing fimbriae sheared from the bacterial surface. The *fimA* gene is heterogeneous, with at least six different fimbrial types of FimA (type I to V and Ib) known [36]–[38] and the antigenic molecular determinants of various types cross-react poorly or not at all [39], [40]. Therefore, whether fimbriae are good immunogens for a vaccine antigen is controversial; it may be appropriate to use “fimbriae-free” MVs as a vaccine immunogen. Further immunological studies using highly purified materials are needed to identify suitable vaccine immunogens to protect against *P. gingivalis* infection.

In the course of the competitive binding assay using mixtures of MV preparations from different species, we unexpectedly identified the neisserial PorB protein among the typical protein bands of *P. gingivalis* MVs in the elution fraction (Fig. 5A and S3). However, PorB was completely unable to bind to the SBs in the absence of *P. gingivalis* MVs (Fig. 5B and 3). Neisserial PorB is a unique porin protein in that it is capable of translocating vectorially into host cell membranes [41], [42] and mitochondria [43]. It is therefore expected that the neisserial PorB derived from the MVs can translocate to the MVs of *P. gingivalis* in a similar manner to the reported translocation into mitochondria or host cell membranes. However, it is notable that the binding of PorB to MVs occurred in 20 mM Tris-Cl buffer alone, i.e., in the absence of any energy sources such as ATP. It is unknown whether PorB can also translocate to MVs of other species besides *P. gingivalis* or to the outer membrane of live *P. gingivalis*. The physiological significance and the mechanism driving this phenomenon are also unknown. Further investigation is needed and the novel approach using SB-Epoxy to obtain ultrapurified *P. gingivalis* MVs may provide clues into as yet undiscovered molecular crosstalk among different bacterial species mediated by MVs.

## Supporting Information

Figure S1 **Binding to SB-Epoxy of MVs from different** ***P. gingivalis*** **strains.** Shown are results of assays using crude MV preparations from the following four different *P. gingivalis* strains: FDC381, W50, YH522, and W83. Crude MVs from a single strain were incubated with the SB-Epoxy (Crude). Unbound components were collected in five washes (Unbound). For MVs of FDC381, W50, and YH522, the bound components were eventually eluted with SDS-PAGE loading buffer (Elu). For MVs of W83, the bound components were eluted twice with a mild denaturation buffer containing 2 M urea (Urea). After one wash with PBS, the components still bound to the SB-Epoxy (even after urea treatment) were treated with 4% CHAPS buffer (CHAPS). “MW” denotes the molecular weight marker. The same volume (10 µl) was applied to each well of each PAGE gel.
(TIF)

Figure S2 **Binding to SB-Epoxy of MVs from two additional periodontopathic bacteria.** Shown are results of assays using crude MV preparations from the following two species of periodontopathic bacteria: *A. actinomycetemcomitans*, strains Y4 and ATCC29522; and *F. nucleatum*, strains ATCC 23726 and #20. Crude MVs from a single strain was incubated with the SB-Epoxy (Crude). Unbound components were collected in five washes (Unbound). The bound components were eventually eluted with SDS-PAGE loading buffer (Elu). “MW” denotes the molecular weight marker. The sample volume (10 µl) was applied to each well of each PAGE gel.
(TIF)

Figure S3 **Competitive binding assay using MVs of** ***P. gingivalis*** **and** ***N. meningitidis*** **.** Shown are results of competitive binding assays of crude MVs of *P. gingivalis* ATCC 33277 and *N. meningitidis* H44/76. (A–C) Crude MVs from a single strain of *P. gingivalis* (A) or *N. meningitidis* (B), and a mixture of *P. gingivalis* and *N. meningitidis* (C) were incubated with SB-Epoxy. Unbound components were collected in five washes with PBS and bound components were eventually eluted with SDS-PAGE loading buffer. Lanes denoted “1” are the starting material of crude MVs from conventional purification (Crude). Lanes denoted “2” are the first unbound fractions (Unbound, 1^st^). Lanes denoted “3” are the fifth unbound fractions (Unbound, 5^th^). Lanes denoted “4” are the elution fractions (Elu). The same sample volume (10 µl) was applied to each well of the PAGE gel. *P. gingivalis* FimA is denoted by an asterisk (*). *N. meningitidis* PorB is denoted by a dagger (†).
(TIF)

Video S1 **Morphological changes and movement of cells in the presence of MVs.** Time-lapse observation of a monolayer of Ca9.22 cells was monitored by an optical microscope (Olympus) equipped with a CCD camera (Andor) in the presence of highly purified MVs isolated by the SB technique. The protein concentration of MVs used in the assay was 20 µg/ml. Images were taken at 30 sec/frame for 30 min immediately after addition of MVs suspended in Eagle’s balanced salt solution (EBSS). The constructed time-lapse images are shown at 100 msec/frame (300 times the original speed). The width of the image is 170 µm.
(AVI)

Video S2 **Morphological changes and movement of cells in the absence of MVs (a negative control).** Time-lapse observation of a monolayer of Ca9.22 cells was monitored in EBSS in the absence of MVs. Images were taken at 30 sec/frame for 30 min. The constructed time-lapse images are shown at 100 msec/frame (300 times the original speed). The width of the image is 170 µm.
(AVI)
