# Supplementary figures and images for: A Novel Approach for Purification and Selective Capture of Membrane Vesicles of the Periodontopathic Bacterium, Porphyromonas gingivalis: Membrane Vesicles Bind to Magnetic Beads Coated with Epoxy Groups in a Noncovalent, Species-Specific Manner
Source: PLoS One. 2014 May 15;9(5):e95137. doi: 10.1371/journal.pone.0095137 (PMC4022494; doi:10.1371/journal.pone.0095137)

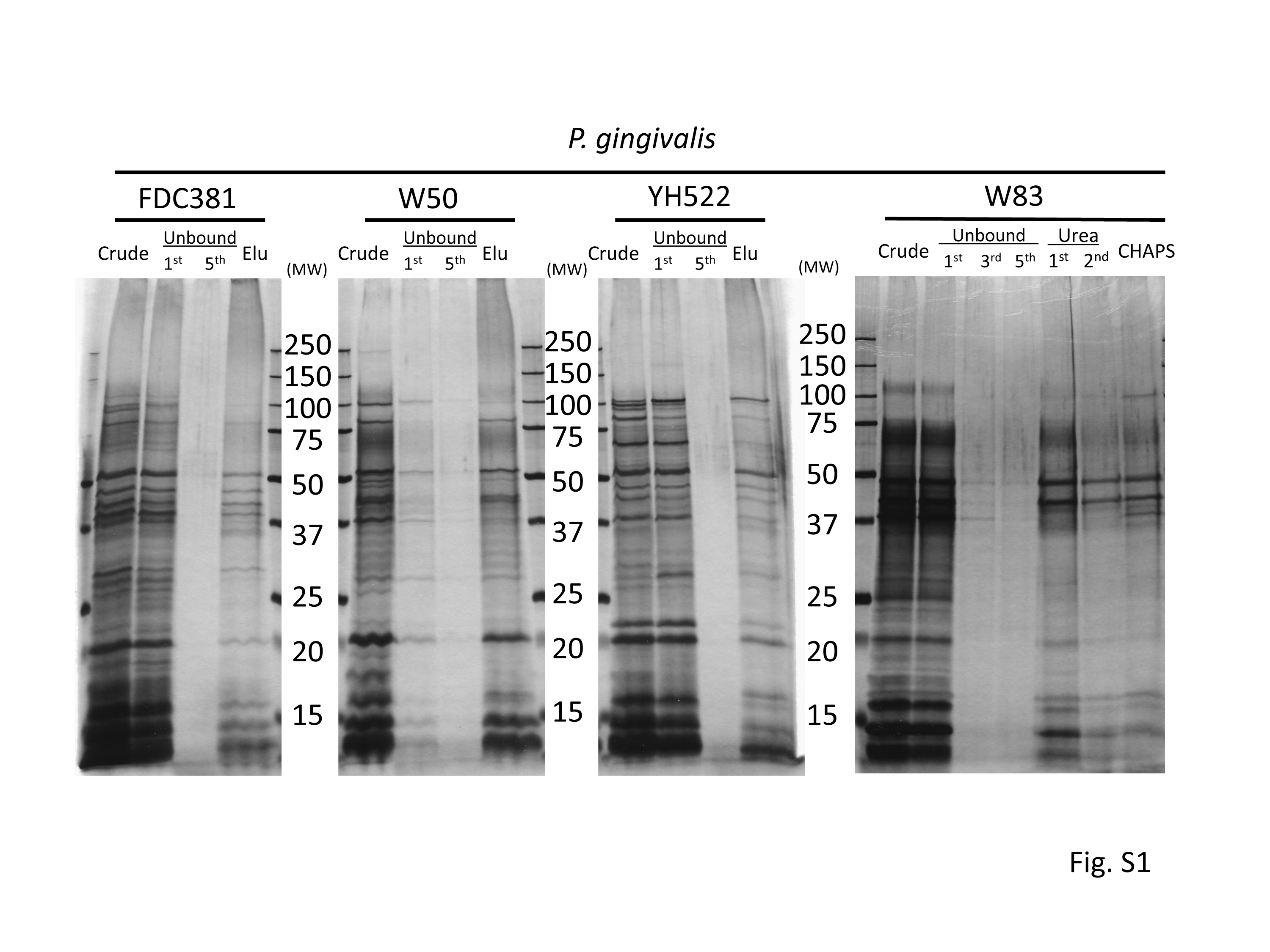

Supplement: Figure S1 — Binding to SB-Epoxy of MVs from different P. gingivalis strains. Shown are results of assays using crude MV preparations from the following four different P. gingivalis strains: FDC381, W50, YH522, and W83. Crude MVs from a single strain were incubated with the SB-Epoxy (Crude). Unbound components were collected in five washes (Unbound). For MVs of FDC381, W50, and YH522, the bound components were eventually eluted with SDS-PAGE loading buffer (Elu). For MVs of W83, the bound components were eluted twice with a mild denaturation buffer containing 2 M urea (Urea). After one wash with PBS, the components still bound to the SB-Epoxy (even after urea treatment) were treated with 4% CHAPS buffer (CHAPS). “MW” denotes the molecular weight marker. The same volume (10 µl) was applied to each well of each PAGE gel. (TIF) [file pone.0095137.s001.tif]

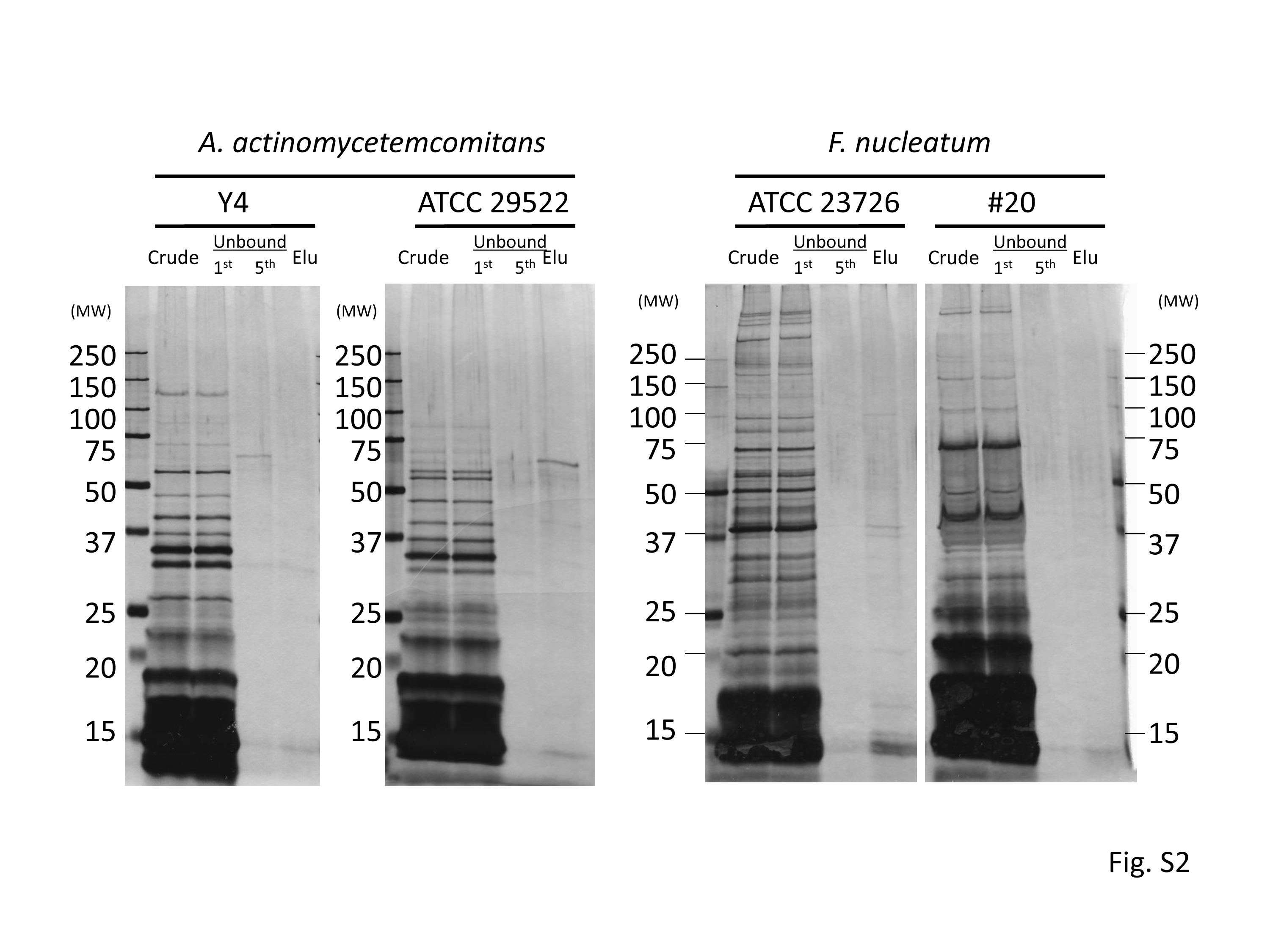

Supplement: Figure S2 — Binding to SB-Epoxy of MVs from two additional periodontopathic bacteria. Shown are results of assays using crude MV preparations from the following two species of periodontopathic bacteria: A. actinomycetemcomitans, strains Y4 and ATCC29522; and F. nucleatum, strains ATCC 23726 and #20. Crude MVs from a single strain was incubated with the SB-Epoxy (Crude). Unbound components were collected in five washes (Unbound). The bound components were eventually eluted with SDS-PAGE loading buffer (Elu). “MW” denotes the molecular weight marker. The sample volume (10 µl) was applied to each well of each PAGE gel. (TIF) [file pone.0095137.s002.tif]

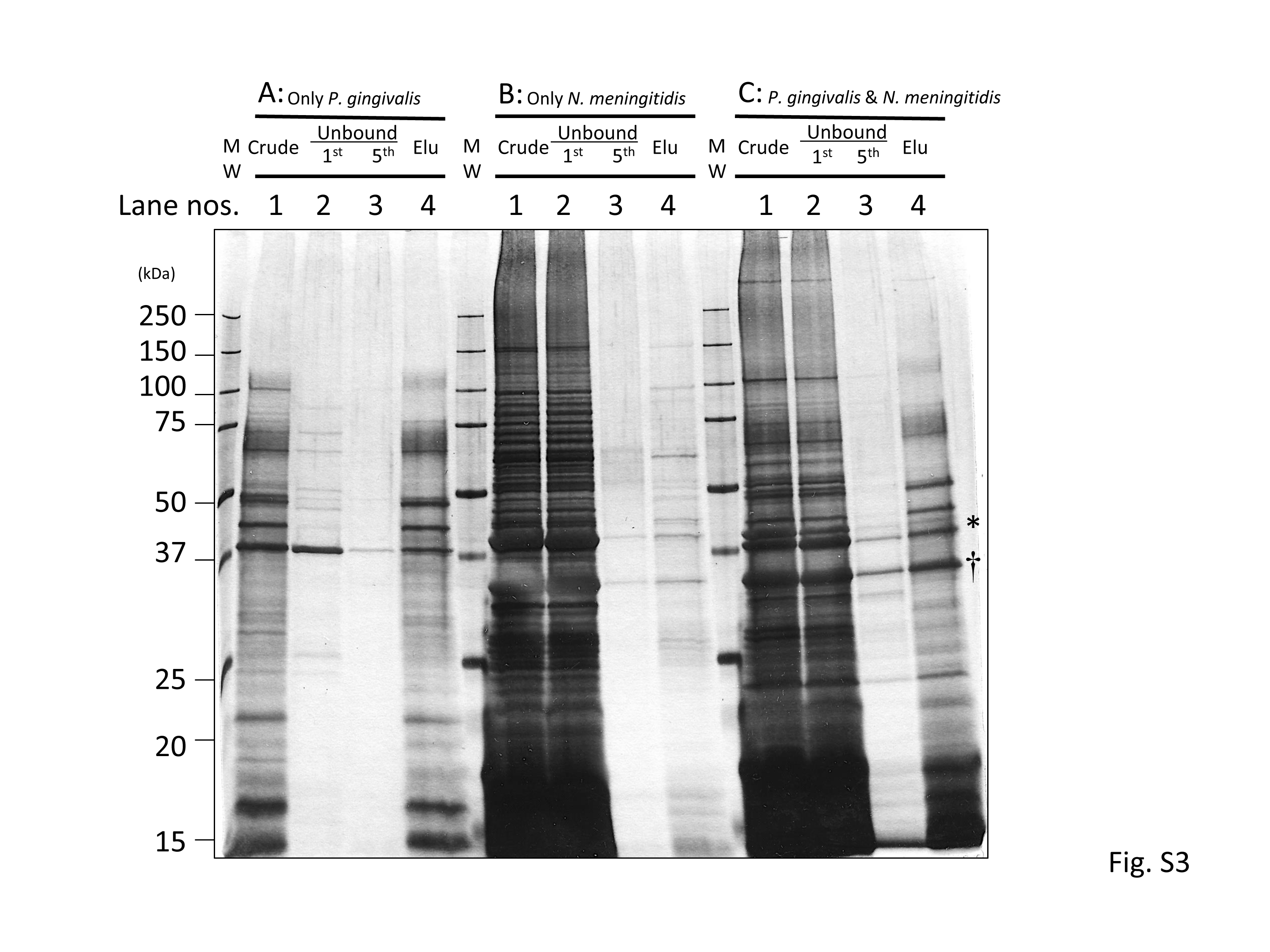

Supplement: Figure S3 — Competitive binding assay using MVs of P. gingivalis and N. meningitidis . Shown are results of competitive binding assays of crude MVs of P. gingivalis ATCC 33277 and N. meningitidis H44/76. (A–C) Crude MVs from a single strain of P. gingivalis (A) or N. meningitidis (B), and a mixture of P. gingivalis and N. meningitidis (C) were incubated with SB-Epoxy. Unbound components were collected in five washes with PBS and bound components were eventually eluted with SDS-PAGE loading buffer. Lanes denoted “1” are the starting material of crude MVs from conventional purification (Crude). Lanes denoted “2” are the first unbound fractions (Unbound, 1st). Lanes denoted “3” are the fifth unbound fractions (Unbound, 5th). Lanes denoted “4” are the elution fractions (Elu). The same sample volume (10 µl) was applied to each well of the PAGE gel. P. gingivalis FimA is denoted by an asterisk (*). N. meningitidis PorB is denoted by a dagger (†). (TIF) [file pone.0095137.s003.tif]
